# A negative selection heuristic to predict new transcriptional targets

**DOI:** 10.1186/1471-2105-14-S1-S3

**Published:** 2013-01-14

**Authors:** Luigi Cerulo, Vincenzo Paduano, Pietro Zoppoli, Michele Ceccarelli

**Affiliations:** 1 Department of Science, University of Sannio, Benevento, Italy; 2 BioGeM s.c.a r.l., Institute of Genetic Research "Gaetano Salvatore", Ariano Irpino (AV), Italy; 3 Institute for Cancer Genetics, Columbia University, New York, NY, USA

## Abstract

**Background:**

Supervised machine learning approaches have been recently adopted in the inference of transcriptional targets from high throughput trascriptomic and proteomic data showing major improvements from with respect to the state of the art of reverse gene regulatory network methods. Beside traditional unsupervised techniques, a supervised classifier learns, from known examples, a function that is able to recognize new relationships for new data. In the context of gene regulatory inference a supervised classifier is coerced to learn from positive and unlabeled examples, as the counter negative examples are unavailable or hard to collect. Such a condition could limit the performance of the classifier especially when the amount of training examples is low.

**Results:**

In this paper we improve the supervised identification of transcriptional targets by selecting reliable counter negative examples from the unlabeled set. We introduce an heuristic based on the known topology of transcriptional networks that in fact restores the conventional positive/negative training condition and shows a significant improvement of the classification performance. We empirically evaluate the proposed heuristic with the experimental datasets of Escherichia coli and show an example of application in the prediction of BCL6 direct core targets in normal germinal center human B cells obtaining a precision of 60%.

**Conclusions:**

The availability of only positive examples in learning transcriptional relationships negatively affects the performance of supervised classifiers. We show that the selection of reliable negative examples, a practice adopted in text mining approaches, improves the performance of such classifiers opening new perspectives in the identification of new transcriptional targets.

## Background

An important challenge of computational biology is the reconstruction of large biological networks from high throughput genomic and proteomic data. Biological networks are used to represent and model molecular interactions between biological entities, such as genes and proteins in a given biological context.

In this paper we focus on the identification of new transcriptional targets, *i.e*. coding DNA regions directly regulated by transcription-factors. Transcription factors are proteins, coded by specific genes, that, alone or with other proteins in a complex, bind the targets cis-regulatory regions and control the target transcriptional activity by promoting or blocking the recruitment of RNA polymerase.

In identifying the interactions between transcription-factors and genes from experimental data, two broad classes of computational methods can be distinguished in literature [1,2]: those that rely on the physical interaction between molecules (gene-to-sequence interaction) which relate transcription factors to sequence motifs found in promoter regions; and algorithms based on the influence interaction that try to relate the expression of a gene to the expression of the other genes in the cell (gene-to-gene interaction). Most of the approaches of the second class are basically unsupervised and model the reconstruction of transcriptional relationships as a classification problem, where the basic decision is the presence or absence of a relationship between a given pair of genes [3-6]. Those methods can be distinguished in: i) *gene relevance network *models, which detect gene-gene interactions with a similarity measure and a threshold, such as ARACNE [7], TimeDelay-ARACNE [8], and CLR [9] that infer the network structure with a statistical score derived from the mutual information and a set of pruning heuristics; ii) *boolean network *models, which adopt a binary variable to represent the state of a gene activity and a directed graph, where edges are represented by boolean functions (e.g. REVEAL [10]); iii) *differential and difference equation *models, which describe gene expression changes as a function of the expression level of other genes with a set of ordinary differential equations (ODE) [11]; and iv) *Bayesian *models, or more generally graphical models, which adopt Bayes rules and consider gene expressions as random variables [12].

The experimental validation of predicted transcriptional regulations is performed with ChIP-on-chip [13], a technique used to investigate interactions between proteins and DNA in vivo by combining chromatin immuno-precipitation (ChIP) with microarray technology (chip). Specifically, it allows the identification of the cistrome, sum of binding sites, for DNA-binding proteins on a genome-wide basis. Whole-genome analysis can be performed to determine the locations of binding sites for almost any protein of interest, in particular transcription factors. The goal of ChIP-on-chip is to localize protein binding sites that may help identify functional elements in the genome. For example, in the case of a transcription factor as a protein of interest, one can determine its transcription factor binding sites throughout the genome.

A recent trend in computational biology aims reconstruct large biological networks with supervised approaches [5,6,14]. Supervised methods require a *training set*, which in our context means a set of transcriptional targets where the information that they are regulated by a transcription factor is known in advance. Training targets are used to estimate a function that is able to discriminate whether a new transcriptional interaction exists. The literature of machine learning proposed several supervised algorithms: neural networks, decision tree, logistic models, and Support Vector Machines (SVM) [15]. Among all SVM gave promising results in the reconstruction of biological networks [16-18]. For example, SIRENE adopted an SVM classifier to reconstruct the regulatory network of *Escherichia coli*, and obtained more accurate results than unsupervised methods based on mutual information (e.g. CLR and ARACNE) [16]. Compared to unsupervised methods, supervised methods are potentially more accurate, but in fact they need an initial set of known regulatory connections. This is in principle not a restriction as many regulations are progressively discovered and shared among researchers through public regulatory databases. Some examples are: RegulonDB (http://regulondb.ccg.unam.mx), KEGG (http://www.genome.jp/kegg/), TRRD (http://wwwmgs.bionet.nsc.ru/mgs/gnw), Transfac (http://www.gene-regulation.com), IPA (http://www.ingenuity.com).

In general a supervised binary classifier needs both positive and negative examples to learn effectively. In the context of gene regulatory networks this condition is not satisfied, as counter negative examples are not available or may be hard to collect. In functional genomics, information about negative examples is in fact not available, as the input is usually a finite list of genes known to have a given function or to be associated to a given disease, and the goal is to identify new genes sharing the same property. Thus, under a machine learning perspective, the supervised inference of new transcriptional targets falls into a class of semi-supervised learning problems that consists of learning from positive and unlabeled data. The training set is composed just by known positive examples (*positive set*) and the goal is to predict the unknown positive in examples the *unlabeled set*.

Learning from only positive and unlabeled data is a hot topic in the literature of data mining, where three main families of approaches can be distinguished [19]: i) methods that reduce the problem to the traditional two-class learning by selecting reliable negative examples from the unlabeled set [20-25]; ii) methods that do not need labeled negative examples and basically adjust the probability of being positive estimated by a traditional classifier trained with positive and unlabeled examples [14,26]; and iii) methods that treat the unlabeled set as noisy negative examples [27].

In this paper we focus on the first class of approaches that rely on a starting selection of negative examples. The main problem is that some of the selected negative examples could in fact be positives embedded in the unlabeled data, reducing the prediction capability of a binary classifier. We empirically evaluate this effect by simulating the positive contamination inside the negative training set showing that the performance of the classifier improves when the positive contamination is low. Such a result demands for an approach that is able to generate a sufficiently large negative training set without positive contamination.

We propose, NOIT (NOn Indirect Targets), a method to select reliable negative training examples by exploiting the known gene regulatory network topology in the specific context of prediction new transcriptional targets. The method is an extension, to a specific context, of approaches recently published in [28] and [29] where reliable negatives selection benefits from the over presence, in the current known gene regulatory networks, of typical network motifs [30]. We introduce a new heuristic that still exploits the known regulatory network topology but not in terms of network motifs as in the specific context of transcriptional target prediction the relationships between transcription-factors and their targets does not exhibit significant network patterns. The NOIT method gives less importance to indirect targets, i.e. targets of a transcription-factor regulated indirectly through other transcription-factors. The idea is based on the observation that genes controlled directly by a transcription factor or indirectly through other transcription factors are likely to attain for the same family of functions, thus representing unreliable negative candidates. This is supported by the fact that transcription factors evolved in the service of specific biological functions and are usually classified according to their regulatory function [31] and sequence similarity [32,33]. Moreover downstream targets activity is usually modulated by regulatory circuits involving small groups of transcription factors organized in typical network motifs.

We compare NOIT with other negative selection approaches known in literature. For this purpose we adopt the dataset of *Escherichia coli*, where almost all transcriptional regulations are known and a huge amount of experimental data is available for benchmarking (e.g. Faith *et al. *[34]). Furthermore we provide an example of application in the prediction of BCL6 direct targets in normal germinal center human B cells by adopting the results of Basso *et al. *[13] showing that NOIT predicts 29 correct targets in the top 50 ranked genes, outperforming other supervised and unsupervised methods that predict less than 10 correct targets. The paper is organized as follows. The next section (Methods) introduces the NOIT heuristic, overviews the literature methods that are based on a reliable negative selection procedure, and describes the empirical procedures aimed at evaluating the performance of the negative selection heuristics. Section on results reports and discusses the outcomes of the study, and the last section concludes the paper outlining directions for future work.

## Methods

### Problem formulation

In a binary classification problem, given a set of training examples, (*x*_1_, *y*_1_), (*x*_2_, *y*_2_), ..., (*x_n_, y_n_*) ∈ *X *× {+1,-1}, the goal is to determine a function *f*(*x*): *X *→ {-1,+1} that is able to predict the label *y *∈{+1,-1}of a new observation *x *∈ *X*. Machine learning algorithms infer an estimate of the function *f *from the available examples. To distinguish effectively whether a new observation is positive or negative, the training set should contain a sufficient number of both positive and negative examples. Such a conventional condition does not hold in the problem we aim to formalize as the training set is composed by only positive examples. In the context of transcriptional target prediction negative counter examples are in principle not available as the nonexistence of a transcriptional activity is hard to be experimentally verified. Liu *et al. *[20] theoretically showed that a statistical classifier may take advantage from unlabeled examples, and that if the sample size is large enough, the classifier could converge to a good classifier by maximizing the number of unlabeled examples classified as negative while constraining the positive examples to be correctly classified. The selection of reliable negatives from the unlabeled set could be crucial for the quality of a positive only classifier. With those examples a classifier could be trained with a traditional two-class set under the control of a convergence condition. The selection of reliable negative training examples may, or may not, exploit the underlying application domain. For example, in the classification of web documents, reliable negative documents are those that do not contain any of the most frequent words extracted from known positive documents [35].

We propose, NOIT (NOn Indirect Targets), a negative selection heuristic that exploits the known regulatory network topology by giving less importance to indirect targets, and formalized as follows. Let *G *be the set of all genes in an organism and *TF *⊂ *G *the set of transcription factors. Given a transcription factor *tf_i _*∈ *TF*, the goal is to infer a function, $f_{tf_{i}}\left(\phi \left(g\right)\right):G\to \left\{-1,+1\right\}$, from a set of target genes, $P_{tf_{i}}=\left\{\left(g_{1},+1\right),\left(g_{2},+1\right),...,\;\left(g_{n},+1\right)\right\}\subset \left(G\backslash TF\right)$, that are known to be regulated directly by *tf_i _*(i.e. positive examples). The function should be able to predict the label *y *of a new gene $g\in U_{tf_{i}}=G\backslash \left(TF\cup P_{tf_{i}}\right)$ from the unlabeled set. The transformation function *ϕ *describes each gene with an *m*-dimensional real valued feature vector, $\phi \left(g\right):G\to {\mathbb{R}}^{m}$, such as expression values measured in *m *different experimental conditions.

The goal of a negative selection heuristic is to select from the unlabeled set $U_{tf_{i}}$ a sufficiently large negative training set without positive contamination. Our aim is to propose a method based on the assumption that an unlabeled gene $g\in U_{tf_{i}}$ is a bad negative candidate if it is indirectly controlled by *tf_i_*, through other transcription factors. Such information can be extracted from the known gene regulatory network, or in the situation wherein such information is not available, it could be estimated with binding site promoter analysis [32] and/or unsupervised gene regulatory prediction [7,9].

**Figure 1:**
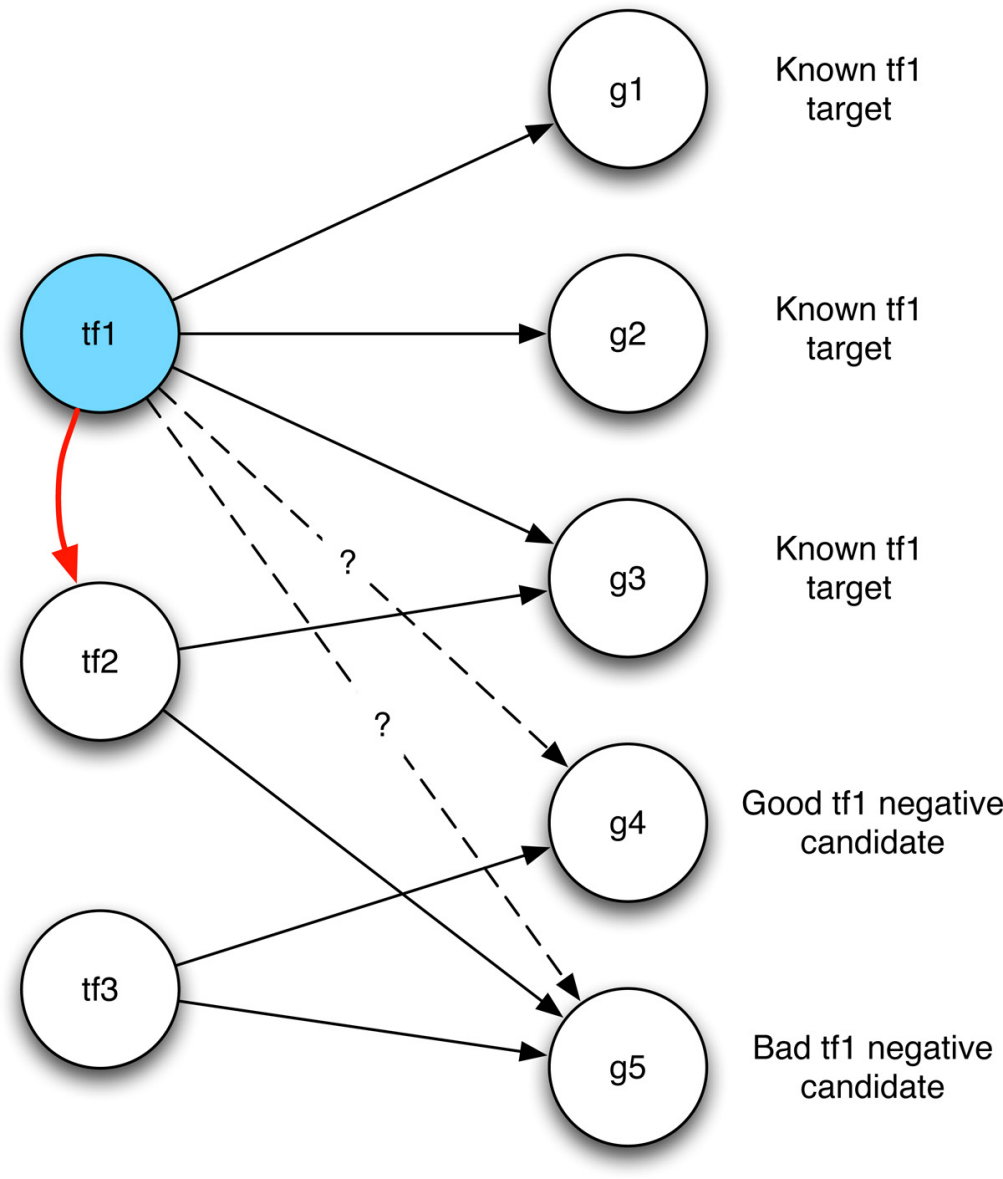
**The NOIT (NOn Indirect Targets) negative selection heuristic**. Reliable negative examples are sampled from the unlabeled set distributed according to an heuristic that promotes the non existence of indirect relationships with the current transcription factor.

**Figure 2:**
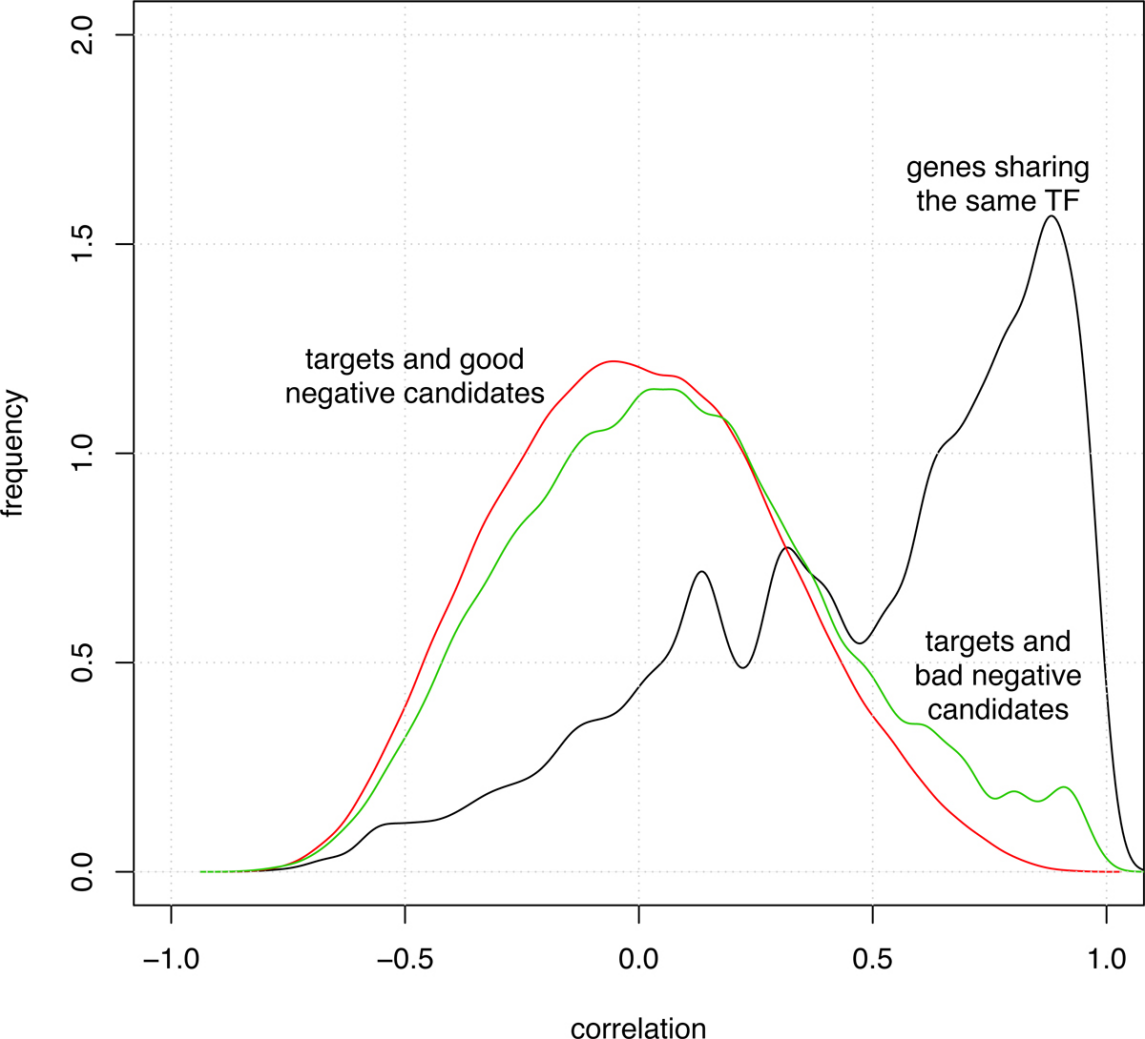
**Distribution of correlation between gene expression profiles**. The distribution of gene expression profiles correlation computed between genes regulated by the same transcription factor (black curve), between targets and good negative candidates (red curve), and between targets and bad negative candidates (green curve).

**Figure 3:**
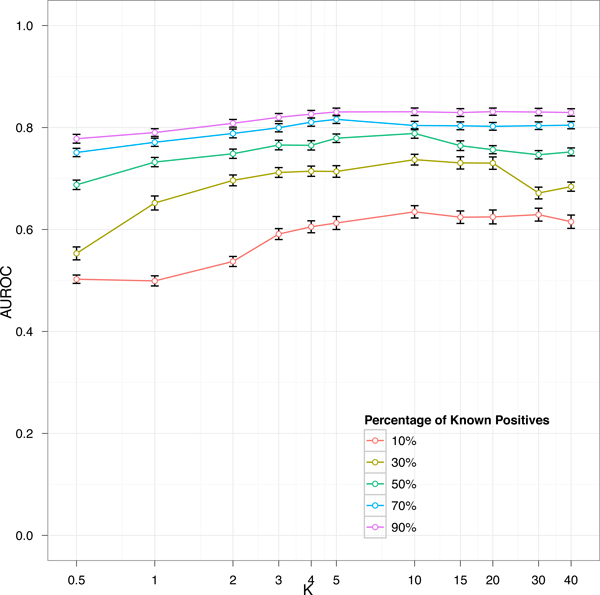
**Effect of the NOIT parameter K on classifier performance**. The parameter K determines the amount of negative candidates that will be included in the training set. The figure shows the classifier performance in terms of AUROC for different values of K. Each curve refers to a different percentage of known positives. The optimal value can be observed around K = 10.

We introduce a probability mass function $pmf_{tf_{i}}\left(g\right)$ of negative candidates distribution to estimate the probability that an example $g\in U_{tf_{i}}$ is a good negative candidate. We compute $pmf_{tf_{i}}\left(g\right)$ as:

$$pmf_{tfi}(g)=\frac{1}{\left|U_{tfi}\right|}\frac{k}{\left|TF\right|}$$

where *k *∈ [1, |*TF*|] is the minimum number of transcription factors, *tf_i_*_+1_, *tf_i_*_+2_, ..., *tf_i_*_+_*_k_*, that link *tf_i _*to *g, i.e*. for every *j *= *i*, ..., *i *+ *k *-1, *tf_j_*_+1 _is a known target of *tf_j _*. The term $1/|U_{tf_{i}}|$ serves to scale the probability mass function to sum to 1. When a path linking *tf_i _*and *g *through a set of known transcription factors does not exist, we assume that *k *= |*TF*|. In that case the probability is maximum, instead it is minimum when at least one *tf_k _*exists such that g is regulated by *tf_k _*and *tf_k _*is regulated by *tf_i _*(Figure 1). The hypothesis is that the expression profile of genes regulated by *tf_i _*are more correlated with genes selected as bad negatives than those selected as good negatives. This is confirmed with a bootstrapping experiment where we selected (many times, e.g. 1000) two random genes, *g*_1 _and *g*_2_, belonging to the targets of a transcription factor, and two genes, *g_good _*and *g_bad_*, belonging respectively to good and bad negative candidates as selected by the NOIT procedure. We computed the correlation between *g*_1 _-*g*_2_, *g*_1 _-*g_good_*, and *g*_1 _-*g_bad _*obtaining the three distributions shown in Figure 2. The black curve shows the distribution of correlation between genes within the same targets, the red curve shows the distribution of correlation between targets and bad negative candidates, and the green curve shows the distribution of correlation between targets and bad negative candidates. A two sample Mann-Withney Test between the latter two distributions shows a significant difference (W = 5940280284, p-value < 2.2 × 10^-16^) suggesting that the NOIT procedure is able to select negative that are more distant, in term of correlation, from targets. With a learning scheme similar to SIRENE [16] we divide the unlabeled set *U_tfi _*into three random folds. The labels of each fold are predicted with a binary classifier trained with the known positives and a selection of negative examples drawn from the other two folds. SIRENE adopts a method, known as PU learning (Positive Unlabeled learning), that is strongly affected by the positive contamination of unlabeled examples as all unlabeled examples are considered good negative candidates. We limit such a contamination by selecting the top *NC *negative candidates scored by the above introduced probability mass function *pmf_tfi_*(*g*). We consider a number of negatives candidates, *NC*, depending on the number of known positives $N\;C=K*|P_{tf_{i}}|$. The parameter *K *may affect the performance of the classifier. With an experiment performed in the context of Escherichia Coli we observed on the independent test set that the best performance is obtained with *K *around 10 (Figure 3).

### Negative selection methods in literature

In this Section we briefly review the most important positive only classification methods that include a reliable negative selection step in their classification schema.

#### Spy-SVM

Spy-SVM is a technique proposed in [20] that works as follows. A percentage of known positives, {*s*_1_, *s*_2_, ..., *s_k_*}, randomly selected from $P_{tf_{i}}$, that act as '*spies*', are sent to the unlabeled set $U_{tf_{i}}$. An SVM classification algorithm is trained with positive examples (without the spies) and the unlabeled set (with the spies) assumed as negatives. The spies should behave identically to the unknown positive examples belonging to $U_{tf_{i}}$, and this allows to reliably infer the behavior of the unknown positive examples. A threshold *t *is employed to make the decision whether an example in $U_{tf_{i}}$ is a reliable negative or not. Examples with a probability of being positive, *P*(*f*(*x*) = +1), lower than *t *are the most likely negative examples. The threshold is intuitively calculated as the minimum of the probability of being positive of spies, i.e. *t *= *min*{*P*(*f*(*s*_1_) = +1), *P*(*f*(*s*_2_) = +1), ..., *P*(*f*(*s_k_*) = +1)}. This means that all the spy examples should be classified as positives.

#### PSoL - Positive Sample only Learning

PSoL selects strong negative example using the Euclidean distance measure [21]. The algorithm starts with a negative candidate that is the most farthest unlabeled example from $P_{tf_{i}}$ calculated as the maximum of the minimum distance from the elements of $P_{tf_{i}}$. More negative candidates are selected from the unlabeled set $U_{tf_{i}}$ satisfying the constrain that are different from the known positive examples and farthest from the previously selected negative ones. The algorithm assumes that the negative examples in the unlabeled set are located far from positives and from the previous selected negative examples. The last condition assures that the negative set spans the whole negative examples in the unlabeled set. Given such initial negative set, the PSoL method iteratively expands the negative set by using a two-class SVM trained with known positives and the current negative selection. Negative set expansion is repeated until the size of the remaining unlabeled set goes below a predefined number. At this last step, the unlabeled data points with the largest positive decision function values are declared as the positives.

#### Rocchio-SVM

Rocchio-SVM is based on a technique adopted in information retrieval to improve the recall of pertinent documents through relevance feedback [22]. It identifies the set of reliable negatives by adopting two prototypes, one for the positive class, *c^P^*, and one for the unlabeled ones, *c^U^*, computed as follows:

$$c^{P}=\alpha \frac{1}{\left|P_{tfi}\right|}\overset{}{\underset{g\in P_{tfi}}{\sum }}\frac{\phi \left(g\right)}{\left||\phi \left(g\right)|\right|}-\beta \frac{1}{\left|U_{tfi}\right|}\overset{}{\underset{g\in U_{tfi}}{\sum }}\frac{\phi \left(g\right)}{\left||\phi \left(g\right)|\right|}$$

$$c^{U}=\beta \frac{1}{\left|U_{tfi}\right|}\overset{}{\underset{g\in U_{tfi}}{\sum }}\frac{\phi \left(g\right)}{\left||\phi \left(g\right)|\right|}-\alpha \frac{1}{\left|P_{tfi}\right|}\overset{}{\underset{g\in P_{tfi}}{\sum }}\frac{\phi \left(g\right)}{\left||\phi \left(g\right)|\right|}$$

where *α *and *β *adjust the relative impact of positive and negative training examples. The unlabeled examples that are more similar to the unlabeled prototype than to the positive one, i.e. *sim*(*g, c^P^*) <*sim*(*g, c^U^*), are selected as strong negative examples. To compute such a similarity the Rocchio technique adopts the cosine similarity. With the known positive examples and the selected negative examples a conventional SVM classifier is trained and then used to classify the remaining set of unlabeled examples.

#### Bagging - SVM

Baggin SVM is an ensemble technique that generally improves the performance of individual classifiers when they are unstable or not correlated to each other. Positive only learning have a particular structure that leads to instable classifiers due to the positive contamination of the unlabeled set which can be advantageously exploited by a bagging-like procedure [36,37]. The approach collects the outcome of a huge number classification runs (e.g. 1000), where each classifier, *F_i_*, is trained with the known positive examples, $P_{tf_{i}}$, and a random set of *NC *negative candidates drawn uniformly from $U_{tf_{i}}$, considered as negative examples. The ensemble classifier, *F*, scores an unlabeled example *g *by averaging the scores obtained by that example at each run:

$$F\left(g\right)=\frac{\sum_{{}_{i\in T_{g}}}F_{i}\left(g\right)}{\left|T_{g}\right|}$$

where *g *is a member drawn from $U_{tf_{i}}$, *F_i _*is the *i*-th classifier, and *T_g _*is the set of partial classifiers that were not trained with *g*, i.e. the unlabeled example *g *was not drawn by the random selection.

### Empirical evaluation methods

In this section we introduce the datasets, the basic learning algorithm, and the methods we adopted to empirically evaluate to which extend a negative selection heuristic improves the performance of a classifier trained to infer new transcriptional targets.

#### Datasets

To test our approach we adopt the well known dataset of *Escherichia coli *provided by Faith *et al. *[34], and a dataset that was adopted by Basso *et al. *[13] to predict BCL6 direct target genes in normal germinal center human B cells.

The dataset of Escherichia coli consists of 445 different Affymetrix Antisense2 microarray expression profiles for 4345 genes. The transcriptional regulatory network of Escherichia coli is the most complete annotated network consisting of 3293 experimentally confirmed relationships between 154 transcription factors and 1211 direct targets extracted from RegulonDB (version 5) [38].

The dataset of Basso *et al*. is deposited in the Gene Expression Omnibus database and is accessible through GEO series accession number GSE12195. It consists of 136 expression profiles of 73 B-cell lymphoma biopsies, 10 purified tonsillar geminal center, 10 naive and memory B cells, 38 Follicular lymphoma biopsies, and 5 lymphoblastoid cell lines. We normalized the dataset from CEL files according to the RMA procedure [39] and filtered out probes with low inter experiment variability by means of the *varFilter *function of the *genefilter *Bioconductor package. The final dataset is composed by 136 samples and 9876 genes. Basso *et al*. identified a group of 120 new core targets down-regulated by BCL6 with an integrated biochemical-computational-functional approach (see Supplemental Table S2 of [13]), validated through ChIP-on-chip.

We show that those 120 new core targets can be predicted with a supervised learning approach starting from a positive training set of 171 targets annotated as down-regulated by BCL6 in a previous work by Ci *et al. *[40]. For the NOIT negative selection procedure we rely on 47 transcription factors known to be regulated by BCL6 by TRANSFAC sequence motifs analysis which considers those that exhibit a BCL6-bound enrichment in their promoter regions as reported in [13]. Their targets were predicted preliminary with ARACNE as reported in the supplemental Table 5 of reference [13].

#### Basic Learning algorithm

We use the Support Vector Machine (SVM), with Platt scaling [41], to estimate the probability that a target is regulated by a transcription-factor. In particular we use the SVM implementation provided by KERNLAB [42], a package for kernel-based machine learning methods in R. The basic element of an SVM algorithm is a kernel function *K*(*x*_1_, *x*_2_), where *x*_1 _and *x*_2 _are feature vectors of two gene targets. The idea is to construct a separation hyperplane between two classes, +1 and -1, such that the distance of the hyperplane to the points closest to it is maximized. The kernel function implicitly maps the original data into some high dimensional feature space, in which the optimal hyperplane can be found. In our experiment we adopt an SVM classifier for each transcription-factor *tf_i _*∈ *TF *trained with the known positive targets and the reliable selection of negative examples performed with a negative selection approach. Such a classifier in then used to score the set of genes *g *∈ *G\TF *according to their probability to be regulated by *tf_i_*. We used C-support vector classification (C-SVC) which solves the following problem:

$$\underset{\alpha }{\text{min}}\frac{1}{2}\alpha^{T}y_{i}y_{j}K\left(x_{i},x_{j}\right)\alpha -{\text{e}}^{T}\alpha$$

subject to: *y^T ^α *= 0, where *y_i _*∈ {+1,-1}is the class of vector *x_i_*, 0 ≤ *α_i _*≤ *C, i *= 1, ..., 2*n*, e is a vector with all elements equal to one, and *K*(*x_i_, y_j_*) is a kernel function. We adopt a radial basis kernel function defined as:

$$K\left(x_{i},x_{j}\right)={e^{-\gamma |x_{i}-x_{j}|}}^{{}^{2}}$$

where *C *and *γ *are parameters that we set empirically inside the training loop [43].

#### Cross validation and performance measures

**Figure 4:**
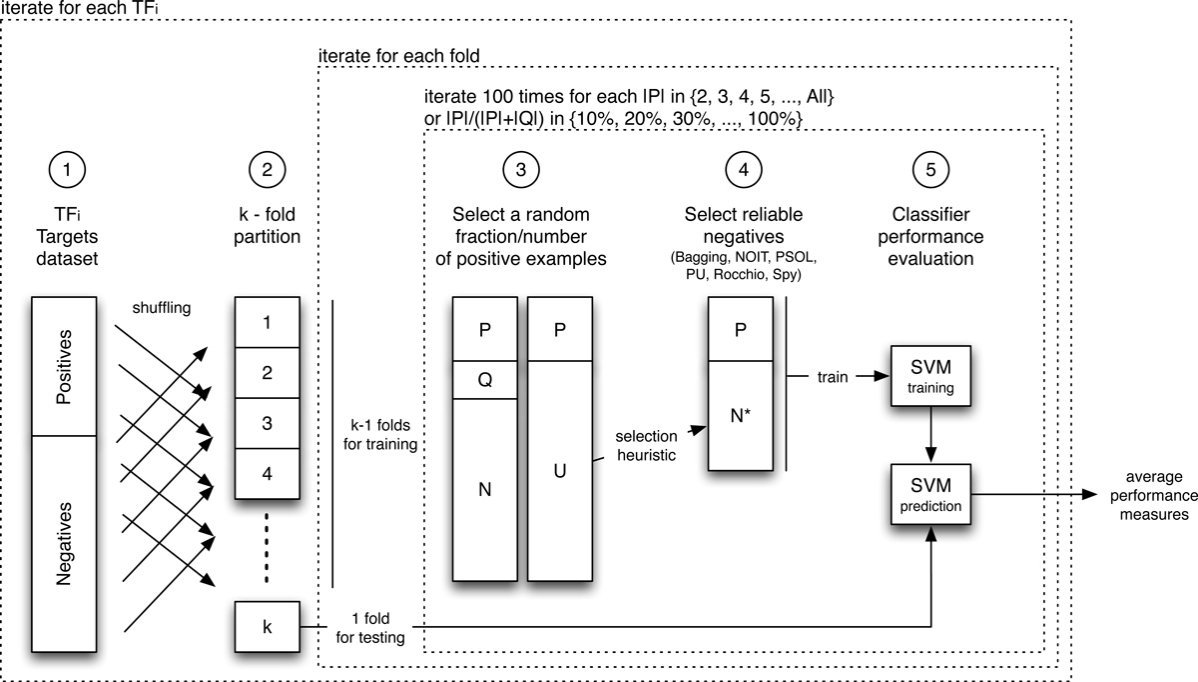
**Evaluation procedure**. A negative selection method is evaluated by adopting a completely labeled dataset and a stratified k-fold cross validation procedure, where the number of known positives is varied linearly starting from 2 or according to its percentage with respect to the unknown positives (from 10% to 100%). To limit the selection bias of known positives, within each k-fold, the percentage of known positives is re-sampled 100 times.

To estimate the unknown performance of a classifier designed for discrimination we adopt a workflow consisting of 5 steps (Figure 4). For each transcription factor *tf_i _*∈ *TF *we partition the original dataset into 10 random folds. Alternatively 9 folds are used for training, while the other fold is used for testing (step 2). Each fold contains a density of positives that is almost similar to the density of positives in the original dataset. The known targets regulated by *tf_i _*belonging to the current training set is split into a positive set $P_{tf_{i}}$, assumed to be the known positive training set, and an unknown set $Q_{tf_{i}}$, forming with $N_{tf_{i}}$ the current unlabeled set $U_{tf_{i}}$ (step 3). The size of $P_{tf_{i}}$ is incremented linearly starting from 2 or according to the fraction $\frac{\left|P_{tf_{i}}\right|}{\left|P_{tf_{i}}\cup Q_{tf_{i}}\right|}$. To limit the selection bias we re-sample $P_{tf_{i}}$ 100 times. The negative training set is extracted from the unlabeled set, $U_{tf_{i}}$ (step 4), and adopted, together with the current known positives, to train an SVM classifier (step 5). Genes belonging to the test set are scored according to the current classifier and the accuracy of classification is evaluated at different ranking levels in terms of precision and recall as follows:

$$PR_{n}=\frac{TP_{n}}{n};\;RC_{n}=\frac{TP_{n}}{\left|targets\left(tf_{i}\right)\right|}$$

where *TP_n _*is the number of true positives appearing in the top *n *ranked targets, and *targets*(*tf_i_*) is the set of *tf_i _*targets we want to predict in each test set. Instead, true positive rates and false positive rates are computed as:

$$T\;P\;R_{n}=\frac{TP_{n}}{\left|Q_{tf_{i}}\right|};\;FPR_{n}=\frac{n-TP_{n}}{\#true\;negatives}$$

where #*true negatives *is the number of true negatives in the test set. From those measures we compute also aggregate performance measures, such as: AUROC (areas under the ROC curve) and AUPR (area under the precision/recall curve). Within a selection of known positives performance measures are averaged among all folds, all positive sampling runs, and all transcription factors obtaining an overall performance estimation of the classifier.

## Results and discussion

### Effect of positive contamination

**Figure 5:**
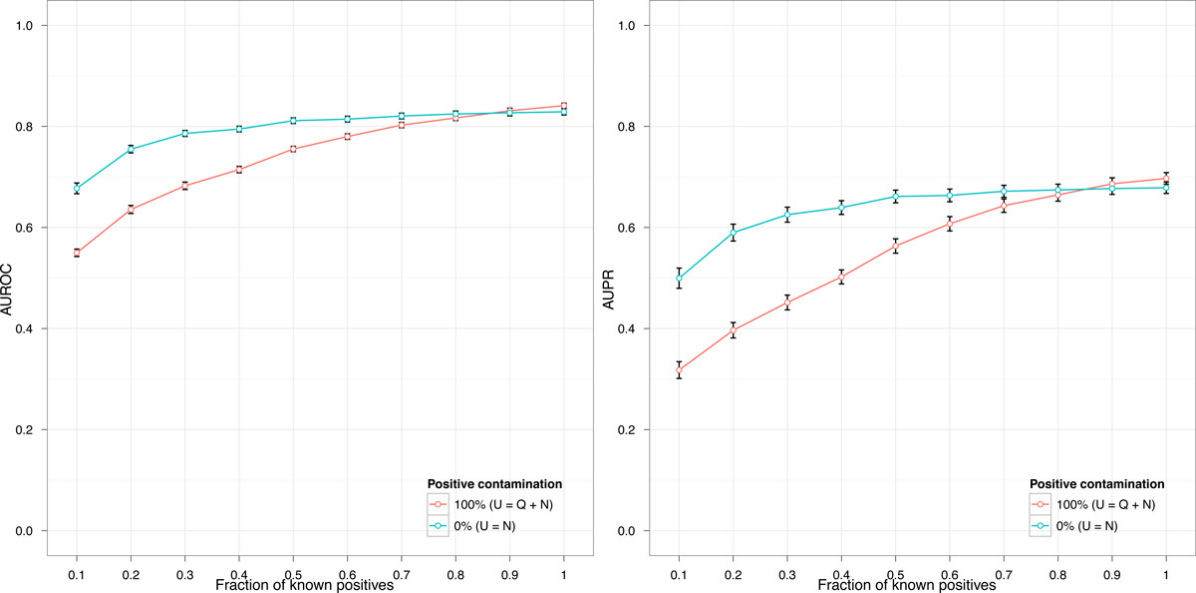
**Effect of positive contamination on classifier performance**. Positive contamination, i.e. the fraction of positives in the unlabeled training set, affects the performance of a classifier. The figure shows two extreme conditions: a classifier trained with unlabeled data totally contaminated with positive examples (100%), and a classifier trained without positive contamination (0%). On the left the performance is shown in terms of AUROC (area under the roc curve), while on the right it is shown in terms of AUPR (area under the precision/recall curve).

The contamination of the training set with positive examples considered wrongly as negatives affects the performance of a classifier. We define the level of positive contamination as the fraction $\frac{\rho }{Q}$ of unknown positives, with respect to the total number of unknown positives (*Q*), selected wrongly as negatives. Figure 5 shows the effect, in terms of AUROC (on the left) and AUPR (on the right), of positive contamination in two extreme conditions: a training set with full positive contamination $\left(\frac{\rho }{Q}=\frac{Q}{Q}=100\%\right)$ and a training set with no positive contamination $\left(\frac{\rho }{Q}=\frac{0}{Q}=0\%\right)$. In the first all unknown positives have been selected (wrongly) as negatives, *U *= *Q *+ *N*. Instead, in the second the training set is composed just by true negatives, *U *= *N *, and represents an ideal classifier with a perfect negative selection heuristic. In principle the actual performance of a negative selection heuristic should be within the area delimited by the two curves.

Both classifiers have been trained in the context of Escherichia coli with the procedure depicted in Figure 4 at different levels of known positives (on the x-axis between 0.1 and 1). The main effect is that the performance of both contaminated and uncontaminated classifiers decreases with the fraction of known positives, although the proportion of that decrement is more rapid for the classifier trained with full positive contamination. When the fraction of known positives is minimum (0.1) the difference between contaminated and uncontaminated classifiers is maximum.

### Effect of the negative selection approach

**Figure 6:**
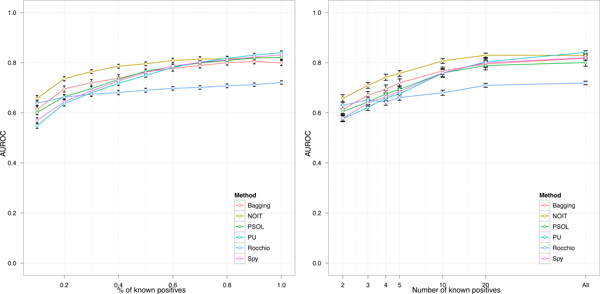
**Classification performance of different negative selection methods**. The performance of different negative selection methods for the prediction of transcriptional targets in Escherichia coli. The figures show the classifier performance in terms of AUROC at different percentage of known positives (on the left), and at different number of known positives (on the right).

The performance of a negative selection approach is affected by the proportion of known positives available in the training set. With the evaluation procedure depicted in Figure 4 we evaluated the performance of a negative selection approach by varying both the relative fraction and the absolute number of known positives. The latter being more in accordance with practical purposes, as users only know the total number of positives which they have. Figure 6 reports, for each method, the average AUROC computed at different fraction of known positives (on the left) and at different number of known positives (in logarithmic scale on the right). On average the performance of each method increases with the quantity of known positives. With the exception of Rocchio each method reaches the maximum performance (AUROC around 0.8) when the training set is completely labeled, i.e. the percentage of known positives is maximum (100%). At low levels of known positives the difference among methods is more significant. Up to a percentage of 60% of known positives, or, up to a number of 20 known positives, in the training set, the NOIT procedure outperforms significantly all other methods. At low levels of known positives the worst performance is registered by PU, as in fact does not adopt any negative selection approach. Instead, at high levels of known positives the worst performance is registered by Rocchio.

Table 1 summarizes the performance of each method in terms of average Recall computed at 60% and 80% of precision. The table reports, at different fraction of known positives, the 95% confidence intervals of Recall measures and the statistical significance (corrected with Benjamini & Hochberg) obtained with a pairwise t-test performed between NOIT and each other method. The adoption of t-test was preliminarly justified as Recall measures follow a normal distribution (Shapiro test, p-value < 2.2 · 10^-16^)and the one-way ANOVA test showed that Recall measures among methods are significantly different (ANOVA, p-value < 2.2 · 10^-16^). At low levels of known positives (precisely at 10% and 30%) the NOIT procedure outperforms significantly all other methods (with the exception of Bagging that exhibits a marginal significant difference when the precision is set to 60%). The increment in Recall can be estimated around 10% with respect to Bagging which is the current state of the art in supervised inference of gene regulatory connections [16,37].

**Table 1:** Recall of negative selection heuristics at 80% and 60% of precision.

Method	%of Known Positives	Recall (Pr = 80%)	p-value (corrected)	Recall (Pr = 60%)	p-value (corrected)
NOIT	10	0.179 (± 0.052)		0.203 (± 0.053)	

PSOL	10	0.043 (± 0.020)	**2.0 · 10^-5^**	0.070 (± 0.031)	**1.2 · 10^-4^**
BAGGING	10	0.066 (± 0.027)	**7.1 · 10^-4^**	0.132 (± 0.051)	9.5 · 10^-2^
ROCCHIO	10	0.036 (± 0.023)	**1.1 · 10^-5^**	0.053 (± 0.032)	**2.0 · 10^-5^**
SPY	10	0.022 (± 0.011)	**7.3 · 10^-7^**	0.038 (± 0.017)	**6.4 · 10^-7^**
PU	10	0.013 (± 0.004)	**2.0 · 10^-7^**	0.038 (± 0.017)	**6.4 · 10^-7^**

NOIT	30	0.252 (± 0.060)		0.384 (± 0.059)	

PSOL	30	0.140 (± 0.039)	**5.9 · 10^-3^**	0.232 (± 0.052)	**5.7 · 10^-4^**
BAGGING	30	0.158 (± 0.047)	**3.5 · 10^-2^**	0.272 (± 0.067)	**2.9 · 10^-2^**
ROCCHIO	30	0.006 (± 0.002)	**1.8 · 10^-10^**	0.010 (± 0.006)	**1.2 · 10^-16^**
SPY	30	0.123 (± 0.036)	**1.1 · 10^-3^**	0.200 (± 0.049)	**2.0 · 10^-5^**
PU	30	0.079 (± 0.024)	**3.3 · 10^-6^**	0.160 (± 0.036)	**3.5 · 10^-8^**

NOIT	50	0.294 (± 0.062)		0.446 (± 0.065)	

PSOL	50	0.240 (± 0.056)	3.6 · 10^-1^	0.366 (± 0.064)	1.3 · 10^-1^
BAGGING	50	0.245 (± 0.053)	3.9 · 10^-1^	0.374 (± 0.069)	1.8 · 10^-1^
ROCCHIO	50	0.010 (± 0.006)	**1.1 · 10^-11^**	0.017 (± 0.011)	**1.3 · 10^-17^**
SPY	50	0.228 (± 0.062)	2.6 · 10^-1^	0.336 (± 0.067)	**4.0 · 10^-2^**
PU	50	0.230 (± 0.053)	2.5 · 10^-1^	0.320 (± 0.056)	**9.8 · 10^-3^**

NOIT	70	0.278 (± 0.064)		0.486 (± 0.066)	

PSOL	70	0.249 (± 0.063)	7.4 · 10^-1^	0.397 (± 0.071)	1.1 · 10^-1^
BAGGING	70	0.304 (± 0.059)	7.4 · 10^-1^	0.433 (± 0.071)	3.7 · 10^-1^
ROCCHIO	70	0.011 (± 0.006)	**1.6 · 10^-10^**	0.019 (± 0.012)	**5.9 · 10^-19^**
SPY	70	0.233 (± 0.064)	5.0 · 10^-1^	0.359 (± 0.074)	**2.6 · 10^-2^**
PU	70	0.305 (± 0.066)	7.4 · 10^-1^	0.435 (± 0.068)	3.7 · 10^-1^

NOIT	90	0.328 (± 0.066)		0.511 (± 0.065)	

PSOL	90	0.239 (± 0.070)	1.4 · 10^-1^	0.391 (± 0.081)	**4.1 · 10^-2^**
BAGGING	90	0.352 (± 0.065)	7.5 · 10^-1^	0.494 (± 0.062)	8.6 · 10^-1^
ROCCHIO	90	0.011 (± 0.005)	**3.7 · 10^-12^**	0.022 (± 0.013)	**4.9 · 10^-20^**
SPY	90	0.296 (± 0.068)	7.4 · 10^-1^	0.436 (± 0.071)	1.8 · 10^-1^
PU	90	0.337 (± 0.067)	1	0.509 (± 0.064)	1

The table shows, at different percentage of known positives, the average Recalls of negative at 80% and 60% of precision (lower and upper 95% confidence intervals is shown in parentheses). The p-value column (corrected with Benjamini & Hochberg) is the outcome of a t-test performed to check whether the recall of NOIT is greater than the recall of another negative selection method. A p-value shown in boldface means that the statistical significance of the test is less than 0.05.

### Prediction of BCL6 core targets in GC human B cells

**Figure 7:**
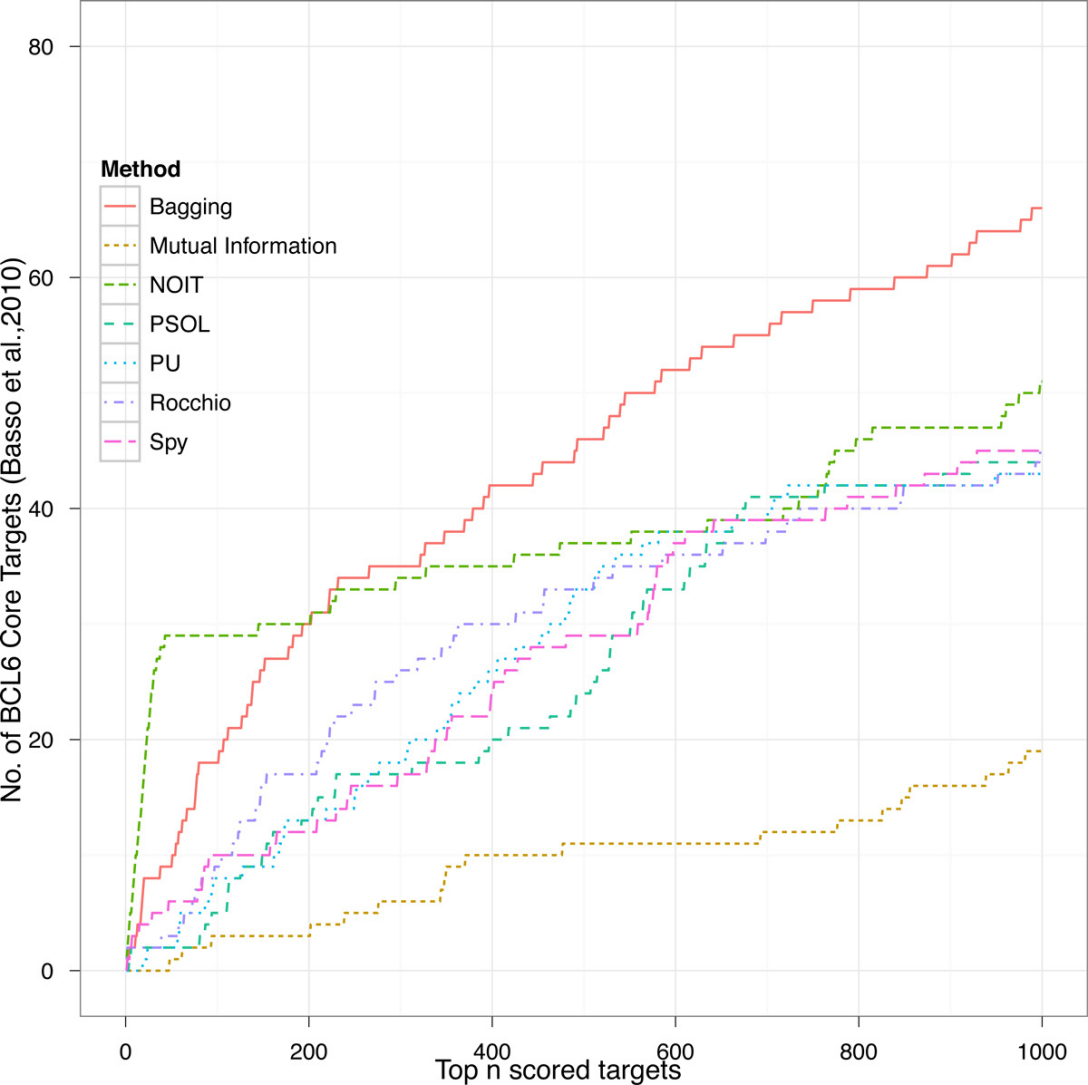
**Top n BCL6 Core targets in GC human B cells predicted with different negative selection methods**. The number of true BCL6 targets as predicted by different negative section procedures and validated with those published by Basso *et al. *[13].

In order to illustrate an examples of application we predict BCL6 core targets in GC human B cells adopting data and results provided by Basso *et al. *[13]. Figure 7 shows the number of true BCL6 core targets appearing in the top *n *genes ranked by an SVM classifier trained with different negative selection approaches. Each classifier has been trained by using the previously known targets provided by Ci *et al. *[40] and the predicted ranked set of genes has been compared with the BCL6 new core targets published by Basso *et al. *[13]. For the NOIT selection procedure we rely on 47 transcription-factors, reported in the Supplemental Table S5 of by Basso *et al. *[13], known to be controlled by BCL6 by means of TRANSFACT sequence motif analysis. The Figure includes also the result obtained with ARACNE [7], an unsupervised method adopted by Basso *et al. *[13], that ranks genes according to their mutual information with BCL6. It is noticeable that supervised reverse engineering methods perform better than unsupervised, a result already confirmed in literature [16]. Instead, among supervised methods there is a remarkable difference in the top 50 ranked genes, where NOIT predicts 29 correct targets (60% precision) outperforming other methods that predict less than 10 correct targets. Over the first 200 ranked genes the Bagging method exhibits the best performance reaching a correct prediction of 66 targets in the first 1000 ranked genes, whereas NOIT predicts only 51 and the others less than 45.

We like to remark that with this experiment we predicted an interesting number of BCL6 targets without the integrated approach consisting of wide spectrum genomics experiments adopted by Basso *et al. *[13] (Figure S6 of [13]). Furthermore, among supervised techniques, the NOIT procedure can take advantage from supplemental transcriptional information, which is aviable in many contexts.

## Conclusions

The availability of only positive examples affects negatively the performance of supervised classifiers. This is particularly manifested in the context of learning transcriptional relationships. We showed that the selection of reliable negative examples, a practice adopted in text mining approaches, could improve the performance of such classifiers opening new perspectives in predicting new transcriptional targets. We introduced a new negative selection heuristic, NOIT, that promotes, as negative candidates of a transcription-factor, genes that are not regulated indirectly through other transcription-factors. The method has been tested against other negative selection procedures showing that it is able to improve the average performance of almost 10%, in terms of AUROC and AUPR, especially when the number of known positives is low. We provided an example of application in the context of prediction of BCL6 direct core targets in normal germinal center human B cells by adopting the results of Basso *et al. *[13]. We showed that in the top 50 genes, ranked with the NOIT method, 29 targets out of 120 are those experimentally demonstrated by Basso *et al. *[13]. This is promising as such targets have been predicted without intersecting the results of ChIP-on-chip assays, ARACNe outcomes, and transcriptional repression in GC experiments.

Threats to external validity, concerning the possibility to generalize our findings, affect the study as we evaluated the heuristics on a limited number of organisms. The study can be replicated as the tools are available upon request to authors and experimental datasets are publicly available.

## Competing interests

The authors declare that they have no competing interests.

## Authors' contributions

LC conceived the negative selection heuristic, designed the empirical evaluation procedure, and drafted the manuscript. VP contributed to the negative selection heuristic definition. PZ contributed to assess the prediction of BCL6 core targets in GC human B cells. MC participated in the coordination of the study and contributed to draft the manuscript. All authors read and approved the final manuscript.

## Declarations

The publication costs for this article were supported by a research project funded by MiUR (Ministero dell'Università e della Ricerca) under grant PRIN2008-20085CH22F.

This article has been published as part of *BMC Bioinformatics *Volume 14 Supplement 1, 2013: Computational Intelligence in Bioinformatics and Biostatistics: new trends from the CIBB conference series. The full contents of the supplement are available online at http://www.biomedcentral.com/bmcbioinformatics/supplements/14/S1.

## References

[B1] Gardner TS Faith JJ Reverse-engineering transcription control networks Physics of Life Reviews 2005 2 65 88 10.1016/j.plrev.2005.01.001 20416858

[B2] Bansal M Belcastro V Ambesi-Impiombato A di Bernardo D How to infer gene networks from expression profiles Molecular Systems Biology 2007 3 78 17299415 10.1038/msb4100120 PMC1828749

[B3] Bansal M Califano A Genome-wide dissection of posttranscriptional and posttranslational interactions Methods Mol Biol 2012 786 131 149 10.1007/978-1-61779-292-2_8 21938624

[B4] Hache H Lehrach H Herwig R Reverse engineering of gene regulatory networks: a comparative study EURASIP J Bioinform Syst Biol 2009 617281 10.1155/2009/617281 PMC3171435 19551137

[B5] Vert JP Reconstruction of Biological Networks by Supervised Machine Learning Approaches 2010 Wiley 163 188

[B6] Grzegorczyk M Husmeier D Werhli AV Reverse engineering gene regulatory networks with various machine learning methods Analysis of Microarray Data 2008

[B7] Margolin AA Nemenman I Basso K Wiggins C Stolovitzky G Dalla Favera R Califano A ARACNE: an algorithm for the reconstruction of gene regulatory networks in a mammalian cellular context BMC Bioinformatics 2006 7 Suppl 1 S7 10.1186/1471-2105-7-S1-S7 16723010 PMC1810318

[B8] Zoppoli P Morganella S Ceccarelli M TimeDelay-ARACNE: reverse engineering of gene networks from time-course data by an information theoretic approach BMC-Bioinformatics 2010 11 154 10.1186/1471-2105-11-154 20338053 PMC2862045

[B9] Faith JJ Hayete B Thaden JT Mogno I Wierzbowski J Cottarel G Kasif S Collins JJ Gardner TS Large-scale mapping and validation of *Escherichia coli* transcriptional regulation from a compendium of expression profiles PLoS Biol 2007 5 e8 10.1371/journal.pbio.0050008 17214507 PMC1764438

[B10] Liang S Fuhrman S Somogyi R Reveal, a general reverse engineering algorithm for inference of genetic network architectures Pac Symp Biocomput 1998 18 29 9697168

[B11] Polynikis A Hogan SJ di Bernardo M Comparing different ODE modelling approaches for gene regulatory networks J Theor Biol 2009 261 511 530 10.1016/j.jtbi.2009.07.040 19665034

[B12] Werhli AV Husmeier D Reconstructing gene regulatory networks with bayesian networks by combining expression data with multiple sources of prior knowledge Stat Appl Genet Mol Biol 2007 6 Article15 17542777 10.2202/1544-6115.1282

[B13] Basso K Saito M Sumazin P Margolin AA Wang K Lim WK Kitagawa Y Schneider C Alvarez MJ Califano A Dalla-Favera R Integrated biochemical and computational approach identifies BCL6 direct target genes controlling multiple pathways in normal germinal center B cells Blood 2010 115 5 975 84 10.1182/blood-2009-06-227017 19965633 PMC2817639

[B14] Cerulo L Elkan C Ceccarelli M Learning gene regulatory networks from only positive and unlabeled data BMC Bioinformatics 2010 11 228 10.1186/1471-2105-11-228 20444264 PMC2887423

[B15] Witten IH Frank E Data mining: practical machine learning tools and techniques 2005 Morgan Kaufmann series in data management systems, Morgan Kaufman

[B16] Mordelet F Vert JP SIRENE: supervised inference of regulatory networks Bioinformatics 2008 24 16 i76 i82 10.1093/bioinformatics/btn273 18689844

[B17] Bock JR Gough DA Predicting protein-protein interactions from primary structure Bioinformatics 2001 17 5 455 460 10.1093/bioinformatics/17.5.455 11331240

[B18] Yamanishi Y Vert JP Kanehisa M Supervised enzyme network inference from the integration of genomic data and chemical information Bioinformatics 2005 21 Suppl 1 i468 i477 10.1093/bioinformatics/bti1012 15961492

[B19] Zhang B Zuo W Learning from positive and unlabeled examples: a survey Information Processing (ISIP), 2008 International Symposiums on 2008 650 654

[B20] Liu B Lee WS Yu PS Li X Partially supervised classification of text documents ICML '02 Proceedings of the Nineteenth International Conference on Machine Learning 2002 San Francisco, CA, USA: Morgan Kaufmann Publishers Inc 387 394

[B21] Wang C Ding C Meraz RF Holbrook SR PSoL: a positive sample only learning algorithm for finding non-coding RNA genes Bioinformatics 2006 22 21 2590 2596 10.1093/bioinformatics/btl441 16945945

[B22] Li X Liu B Learning to classify texts using positive and unlabeled data Proceedings of the 18th international joint conference on Artificial intelligence 2003 IJCAI'03, San Francisco, CA, USA: Morgan Kaufmann Publishers Inc 587 592

[B23] Li XL Liu B Ng SK Learning to identify unexpected instances in the test set Proceedings of the 20th international joint conference on Artifical intelligence 2007 IJCAI'07, San Francisco, CA, USA: Morgan Kaufmann Publishers Inc 2802 2807

[B24] Wang X Xu Z Sha C Ester M Zhou A Semi-supervised learning from only positive and unlabeled data using entropy Proceedings of the 11th international conference on Web-age information management 2010 WAIM'10, Berlin, Heidelberg: Springer-Verlag 668 679

[B25] Li X Liu B Learning to Classify Texts Using Positive and Unlabeled Data IJCAI-03, Proceedings of the Eighteenth International Joint Conference on Artificial Intelligence, Acapulco, Mexico, August 9-15, 2003 2003 587 594

[B26] Elkan C Noto K Learning classifiers from only positive and unlabeled data KDD '08: Proceeding of the 14th ACM SIGKDD international conference on Knowledge discovery and data mining 2008 New York, NY, USA: ACM 213 220

[B27] Liu B Dai Y Li X Lee WS Yu PS Building text classifiers using positive and unlabeled examples ICDM '03: Proceedings of the Third IEEE International Conference on Data Mining 2003 Washington, DC, USA: IEEE Computer Society 179

[B28] Cerulo L Paduano V Zoppoli P Ceccarelli M Labeling negative examples in supervised learning of new gene regulatory connections Computational Intelligence Methods for Bioinformatics and Biostatistics - 7th International Meeting, CIBB, Palermo 2010 159 173

[B29] Ceccarelli M Cerulo L Selection of negative examples in learning gene regulatory networks Bioinformatics and Biomedicine Workshop, 2009. BIBMW 2009. IEEE International Conference on 2009 56 61

[B30] Alon U Network motifs: theory and experimental approaches Nat Rev Genet 2007 8 6 450 461 10.1038/nrg2102 17510665

[B31] Brivanlou AH Darnell JE Signal transduction and the control of gene expression Science 2002 295 5556 813 8 10.1126/science.1066355 11823631

[B32] Matys V Kel-Margoulis OV Fricke E Liebich I Land S Barre-Dirrie A Reuter I Chekmenev D Krull M Hornischer K Voss N Stegmaier P Lewicki-Potapov B Saxel H Kel AE Wingender E TRANSFAC and its module TRANSCompel: transcriptional gene regulation in eukaryotes Nucleic Acids Research 2006 34 Database issue D108 D110 16381825 10.1093/nar/gkj143 PMC1347505

[B33] Stegmaier P Kel AE Wingender E Systematic DNA-binding domain classification of transcription factors Genome informatics. International Conference on Genome Informatics 2004 15 2 276 286 15706513

[B34] Faith JJ Hayete B Thaden JT Mogno I Wierzbowski J Cottarel G Kasif S Collins JJ Gardner TS Large-scale mapping and validation of *Escherichia coli* transcriptional regulation from a compendium of expression profiles PLoS Biol 2007 5 e8 10.1371/journal.pbio.0050008 17214507 PMC1764438

[B35] Yu H Han J chuan Chang KC PEBL: Web Page Classification without Negative Examples IEEE Transactions on Knowledge and Data Engineering 2004 16 70 81 10.1109/TKDE.2004.1264823

[B36] Kim HC Pang S Je HM Kim D Bang SY Support Vector Machine Ensemble with Bagging Proceedings of the First International Workshop on Pattern Recognition with Support Vector Machines 2002 SVM '02, London, UK, UK: Springer-Verlag 397 407

[B37] Mordelet F Vert JP A bagging SVM to learn from positive and unlabeled examples Technical Report http://hal.archives-ouvertes.fr/hal-00523336

[B38] Salgado H Gama-Castro S Peralta-Gil M Díaz-Peredo E Sánchez-Solano F Santos-Zavaleta A Martínez-Flores I Jiménez-Jacinto V Bonavides-Martínez C Segura-Salazar J Mart ìnez-Antonio A Collado-Vides J RegulonDB (version 5.0): *Escherichia coli* K-12 transcriptional regulatory network, operon organization, and growth conditions Nucleic Acids Res 2006 34 Database issue D394 D397 16381895 10.1093/nar/gkj156 PMC1347518

[B39] Irizarry RA Bolstad BM Collin F Cope LM Hobbs B Speed TP Summaries of Affymetrix GeneChip probe level data Nucleic Acids Research 2003 31 4 e15 10.1093/nar/gng015 12582260 PMC150247

[B40] Ci W Polo JM Cerchietti L Shaknovich R Wang L Yang SN Ye K Farinha P Horsman DE Gascoyne RD Elemento O Melnick A The BCL6 transcriptional program features repression of multiple oncogenes in primary B cells and is deregulated in DLBCL Blood 2009 113 22 5536 48 10.1182/blood-2008-12-193037 19307668 PMC2689052

[B41] Lin HT Lin CJ Weng RC A note on Platt's probabilistic outputs for support vector machines Mach Learn 2007 68 3 267 276 10.1007/s10994-007-5018-6

[B42] Karatzoglou A Smola A Hornik K Zeileis A kernlab - An S4 Package for Kernel Methods in R Journal of Statistical Software 2004 11 9 1 20

[B43] Hsu CW Chang CC Lin CJ A practical guide to support vector classification 2003 Department of Computer Science and Information Engineering, National Taiwan University

